# Generalized Joint Hypermobility and Injuries: A Prospective Cohort Study of 185 Pre-Professional Contemporary Dancers

**DOI:** 10.3390/jcm10051007

**Published:** 2021-03-02

**Authors:** Rogier M. van Rijn, Janine H. Stubbe

**Affiliations:** 1 Codarts Rotterdam, University of the Arts, 3012 CC Rotterdam, The Netherlands; jhstubbe@codarts.nl; 2 PErforming Artist and Athlete Research Lab (PEARL), 3012 CC Rotterdam, The Netherlands; 3 Erasmus MC University Medical Center, Department of General Practice, 3015 GD Rotterdam, The Netherlands; 4 Rotterdam Arts and Science Lab (RASL), 3012 CC Rotterdam, The Netherlands

**Keywords:** injury, risk factor, dance, hypermobility, physical examination

## Abstract

Generalized joint hypermobility (GJH) has been mentioned as one of the factors associated with dance injuries, but the findings are inconclusive. This study aims to investigate whether GJH, based on different Beighton score cut-off points, is a potential risk factor for injuries in pre-professional dancers. Four cohorts of first-year pre-professional dancers (*N* = 185), mean age 19.1 ± 1.3 years, were screened on musculoskeletal functioning at the start of their academic year. The Beighton score was used to measure GJH. During the academic year, the dancers completed monthly questionnaires about their physical and mental health. Based on the Oslo Sports Trauma Research Centre Questionnaire on Health Problems (OSTRC), three injury definitions were used (i.e., all complaints, substantial injury, and time-loss injury). To examine potential risk factors for injuries, univariate and multivariate regression models were applied. The response rate of monthly completed questionnaires was 90%. The overall mean (SD) Beighton score was 2.8. The 1-year injury incidence proportion was 67.6% (*n* = 125), 43.2% (*n* = 80), and 54.6% (*n* = 101) for all complaint injuries, substantial injuries, and time-loss injuries, respectively. The multivariate analyses showed a significant association between a previous long lasting injury in the past year and the three injury definitions (*p* < 0.05). Pre-professional contemporary dancers are at high risk for injuries and hypermobility. However, these two variables are not associated with each other. Health professionals should take injury history into account when assessing dance students, because this variable is associated with increased injury risk.

## 1. Introduction

At an early age, dancers participate in long hours of training, classes, and performances to improve their aesthetic, technical, and athletic skills. Artistic, physical, and psychological demands are being pushed when the performance levels of dancers increase [1]. These demands make young dancers prone to injuries, especially during periods of maturation and development [2,3,4]. Injury incident rates within pre-professional and/or contemporary dancers ranged from 0.77 to 4.71 per 1000 h of exposure [3,5]. Insight into the risk factors of dance injuries can give direction to the development of preventive strategies [6,7]. However, a systematic review on risk factors in pre-professional dancers concluded that the level of evidence is scarce [3]. High-quality prospective studies are needed to unravel the multifactorial association between risk factors and dance injuries.

Increased joint flexibility, also known as generalized joint hypermobility (GJH), has been mentioned as one of the factors associated with injuries. GJH is characterized by excessive range of motion in multiple joints that exceeds normal limits, and is assessed by the Beighton score [8,9]. Prevalence of GJH in dance populations ranges between 2% and 44% [10]. This huge variation in prevalences is due to the use of different cut-off points to measure GJH. Although pre-professional dancers with increased joint flexibility are often marked as dancers being “full of potential”, GJH in dancers might have serious disadvantages [11,12]. GJH can be viewed as a sign of vulnerability in terms of physical fitness, psychological distress, and more musculoskeletal complaints (i.e., pain, fatigue) in dancers [13,14]. GJH has been associated with increased risk of (ligament) injuries, recurrent dislocations, knee and ankle effusions, and possible premature arthritis [9,10,15,16]. On the other hand, studies found that GJH was not predictive of injury or total days injured [11,17]. These inconclusive findings might be explained by the lack of high-quality studies [3]. Studies with sufficient power, prospective designs, and uniform injury definitions are needed [3]. Therefore, the objective of this study was to investigate whether GJH, based on different Beighton score cut-off points, is a potential risk factor for injuries in pre-professional dancers.

## 2. Methods

### 2.1. Participants and Procedures

Four cohorts of first-year pre-professional dancers (*N* = 185) of Codarts Rotterdam (University of the Arts) were screened on musculoskeletal functioning at the start of the their academic year in 2016, 2017, 2018, and 2019. During the first month of the academic year, baseline characteristics were recorded including age (years), sex (male/female), educational program (Bachelor Dance or Dance teacher), and injury history (yes/no). Injury history was defined as “any physical complaint resulting in a fulltime loss of dance activities (participation in class, rehearsal, performance practice, and so on) for at least one week beyond the day of onset in the past year” [18,19]. During the academic year, all pre-professional dancers were asked to complete monthly questionnaires about their physical and mental health by using the performing artist and athlete health monitor (PAHM). All pre-professional dancers were informed about the purpose and procedure of this study and provided written informed consent. The study was approved by the Medical Ethics Committee (MEC-2019-0163) of the Erasmus University Medical Center Rotterdam, The Netherlands.

### 2.2. Generalized Joint Hypermobility

Generalized joint hypermobility (GJH) testing was performed during the musculoskeletal screening at the start of the academic year and was done by the physical therapist, specialized in dance medicine, who works at the Performing Arts Health Centre of the university. The Beighton score was used to measure GJH and consists of nine tests with one point allocated for each positive finding: (1) passive dorsiflexion of the fifth metacarpophalangeal joints >90° (one point per side), (2) passive apposition of the thumb to the flexor aspects of the forearm (one point per side), (3) hyperextension of the elbows beyond 10° (one point per side), (4) hyperextension of the knee beyond 10° (one point per side), and (5) forward flexion of the trunk with knees fully extended so that the palms of the hands rest flat on the floor (one point) [8]. The Beighton score was calculated by summing the scores of the five tests, resulting in total scores ranging from 0 (hypomobile) to 9 (hypermobile).

### 2.3. Injury Registration

The Oslo Sport Trauma Research Center (OSTRC) questionnaire is part of the monthly questionnaire and consists of four key questions about the consequences of health problems on participation, training volume, and performance as well as the degree to which the student perceived symptoms. All items ranged from 0 (no problem, no reduction, no effect, or no symptoms) to 25 (cannot participate at all or severe symptoms) [20]. Questions 1 and 4 were scored on a four-point scale (0–8–17–25), while questions 2 and 3 were scored on a five-point scale (0–6–13–19–25). The severity of a health problem was calculated by the sum score of the four questions (scale 0–100) according to the method proposed by Clarsen et al. [20]. If the severity score was higher than zero, a health problem was registered and the student was asked whether the health problem was an injury, mental problem, or other problem. For injuries, the pre-professional dancer was automatically directed to an injury registration form based on an international consensus statement on injury surveillance methodology for football to collect further details (e.g., location, history, and acute or overuse onset) [21]. If a pre-professional dancer reported the same injury as the most severe health problem in two or more consecutive questionnaires, this was counted as one ‘unique’ case of a (fluctuating) problem [5,22].

### 2.4. Injury Definitions

Three definitions of dance related injuries were utilized:

1. All complaints injury: any physical complaint resulting in a severity score higher than zero on the OSTRC questionnaire irrespective of the need for medical attention or time loss from dance activities [23].
2. Substantial injury: any physical complaint resulting in a severity score higher than zero on the OSTRC questionnaire irrespective of the need for medical attention and resulting in problems leading to moderate or severe reductions (value ≥13 on question 2 or 3 of the OSTRC) in training volume or moderate or severe reductions in performance or complete inability to participate in dance [23].
3. Time-loss injury: any physical complaint resulting in a severity score higher than zero on the OSTRC questionnaire and resulting in a dancer not being able to complete a class, rehearsal, or performance or a subsequent class, rehearsal, or performance for one or more days beyond the day of onset [24].

### 2.5. Statistical Analysis

All statistical procedures were performed using SPSS (SPSS, V25.0, IBM, Chicago, IL, USA, 2017) and the statistical significance level was set at an alpha level of <0.05. Descriptive statistics were used to describe the baseline characteristics of all participants using mean values and standard deviation (SD) or number and percentages (%).

The 1-year incidence proportion (IP) of all complaints injuries, substantial injuries, and time-loss injuries was calculated by dividing the number of students who reported at least one injury during the academic year by the number of respondents. The severity of injuries was calculated by the mean (SD) number of full days that a student completely/partly missed their dance activities because of their injury. The characteristics (i.e., location) of injuries were expressed in percentages for the total injuries.

To examine potential risk factors for injuries, univariate and multivariate regression models were applied. Potential risk factors included age (years), sex (female), educational program (Bachelor Dance vs. Bachelor Dance), injury history in the previous year, and generalized joint hypermobility. Analyses were performed in four ways using a different measure of generalized joint hypermobility each time, namely, (1) Beighton score (mean), (2) Beighton cut-off point <3 (not hypermobile), 4–6 (hypermobile), >6 (extreme hypermobile) [25]; (3) Beighton cut-off point ≥4 (hypermobile) [26]; and (4) Beighton cut-off point ≥6 (hypermobile) [26]. First, univariate associations between the potential risk factors and the dichotomized outcome, injured during follow-up (yes/no), were assessed using the three injury definitions. Second, multivariate regression modeling was performed including all potential risk factors and the outcome of interest, resulting in four models (for each measure of GJH) per injury definition. The results of the regression analyses were expressed in odds ratios (ORs) with corresponding 95% confidence interval (95% CI). As logistic regression analyses do not show the proportion of variance that was explained by the model, the Nagelkerke R^2^ was used to express this variance [27]. The Nagelkerke R^2^ varies between −1 and +1. A positive value indicates that, as the value of the risk factor increases, so does the likelihood of incurring an injury. A negative value implies that, as the value of the risk factor increases, the likelihood of the outcome occurring decreases. If a risk factor has a small value of R^2^, it contributes only a small amount to the model [27].

## 3. Results

### 3.1. Participants

All first year students of the Bachelor Dance and Bachelor Dance teacher programs agreed to participate and were consequently included in the present study, resulting in a participation rate of 100%. The cohort comprised 185 students (68.6% females) and the mean age was 19.1 (1.3) years (Table 1). In total, 1665 questionnaires were sent to the students and 1499 were completed, resulting in a response rate of 90.0%.

### 3.2. Generalized Joint Hypermobility

The overall mean (SD) Beighton score for the whole cohort was 2.8 (2.3), with students from the Bachelor Dance program scoring significantly higher than students of the Bachelor Dance teacher program, at 3.2 (2.5) versus 2.2 (2.0), respectively (Table 1). Using a Beighton score cut-off point of 0–3 (not hypermobile), 4–6 (hypermobile), and 7–9 (extreme hypermobile), a total of 25 (13.7%) students were hypermobile and 28 students (15.4%) were extreme hypermobile. No significant difference was found between both Bachelor programs using this cut-off points. Applying the criterion of a Beighton score ≥4/9 resulted in 53 (29.1%) students having GJH. Of these, 40 students were of the Bachelor Dance program versus 13 of the Bachelor Dance teacher program, resulting in a significant difference between both educational programs. Applying the criterion of a Beighton score ≥6/9 resulted in 28 (15.4%) students having GJH. There was a significant difference between the two Bachelor programs in the proportion (19.8% vs. 7.6%) meeting hypermobility at this cut-off point.

### 3.3. Injuries

**Figure 1:**
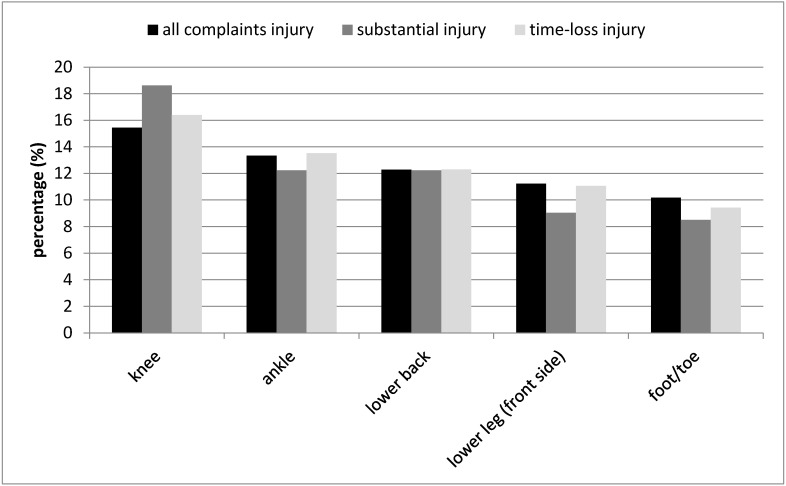
Top five most reported injury locations according to the three injury definitions.

The injury incidence proportion for one academic year was 67.6% (*n* = 125), 43.2% (*n* = 80), and 54.6% (*n* = 101) for all complaint injuries, substantial injuries, and time-loss injuries, respectively. A total of 285 all complaints injuries were reported by 125 students. The mean number of full days a student completely/partly missed dance activities as a result of an all complaint injury was 9.7 ± 19.8 days. A total of 188 substantial injuries were reported by 80 students. The mean number of full days a student completely/partly missed dance activities as a result of an substantial injury was 13.6 ± 23.7 days. A total of 244 time-loss injuries were reported by 101 students. The mean number of full days a student completely/partly missed dance activities as a result of an time-loss injury was 12.0 ± 21.4 days. For all three injury definitions, the most reported injury locations were knee, ankle, lower back, lower leg (front side), and the foot/toe (Figure 1).

### 3.4. Risk Factors

Univariate analyses showed a significant association between a previous long-lasting injury in the past year and all complaints injuries (OR 2.58; 95% CI 1.24–5.35), substantial injuries (OR 3.22; 95% CI 1.69–6.13), and time-loss injuries (OR 3.09; 95% CI 1.59–6.02) during follow-up. Besides, univariate analyses showed that being a student enrolled in the Bachelor Dance teacher program is significantly associated with the occurrence of substantial injuries during follow-up (OR 1.87; 95% CI 1.02–3.42). None of the four operationalized definitions of GJH and none of the other tested variables were univariate associated with the outcomes of interest (Table 2, Table 3 and Table 4). The multivariate analyses showed a significant association between a previous long-lasting injury in the past year and all complaints injuries (range ORs 2.60 to 2.74), substantial injuries (range ORs 2.76 to 2.96), and time-loss injuries (range ORs 2.94 to 3.04) during follow-up. None of the four operationalized definitions of GJH and none of the other tested variables were associated with the outcomes of interest in the multivariate analyses (Table 2, Table 3 and Table 4). The models (for each measure of GJH) explained 5.7% to 6.0% of the variance when using the all complaint injury definition, 11.0% to 11.5% for substantial injuries, and 9.8% to 9.9% when using the time-loss injury definition.

## 4. Discussion

This study investigated whether GJH, measured with the Beighton score, is a risk factor for injuries in pre-professional contemporary dancers. We found an overall mean Beighton score of 2.8 ± 2.3. Using the traditional Beighton cut-off point of 4 or more resulted in a prevalence of GJH of 29.1% (*n* = 53). A more stringent cut-off point (≥6/9) led to a prevalence of 15.4% of the dance students having GJH.

The prevalence of GJH in the current study is within the range of 2% to 81%, as reported in previous studies [10,11,13,16,26,28]. The wide range of reported prevalences can be explained by the lack of consistency in the quality of the studies, different classification and methodology used to measure GJH, and the use of different injury definitions. Therefore, a comparison of the results with the existing literature remains difficult. In a cross sectional study, including 75 pre-professional dancers, a prevalence of GJH of 81% (Beighton cut-off point ≥4/9) and 53% (Beighton cut off point ≥6/9) was reported [26]. Armstrong and colleagues (2019) performed a prospective cohort study among eighty pre-professional [11]. They reported a mean Beighton score of 4.68 ± 1.81 and classified sixty-one dancers (74%) as hypermobile using a Beighton score of ≥4/9. A prospective cohort study by Bronner et al. (2018), in which 180 pre-professional dancers were analyzed, reported a mean Beighton score of 3.59 ± 2.08 [16]. Roussel et al. (2009) found a prevalence of GJH of 44% (Beighton cut-off point ≥4/9) in a population of thirty-two pre-professional dancers. Besides, they reported that 25% presented a Beighton score ranging from 4 to 6 (hypermobile), and 19% were excessively hypermobile (Beighton score 7–9) [29]. Although these studies used the same nine-point Beighton test, they reported a higher prevelance of GJH using the same cut-off points and higher mean Beighton scores compared with our study.

Furthermore, we found a 1-year incidence of all complaints injuries, substantial injuries, and time-loss injuries of 67.6%, 43.2%, and 54.6%, respectively. The results of the multivariate analysis showed that GJH, using one of the Beighton cut-off point variants, was not associated with the occurrence of injuries defined according to the three injury definitions. However, students with an injury history, defined as any physical complaint resulting in a fulltime loss of dance activities for at least 1 week beyond the day of onset in the past year, were more likely to sustain an all complaints injury, a substantial injury, or a time-loss injury.

Our results are in line with those of the prospective cohort study of Roussel et al. (2009) in which GJH was assessed using the Beighton test and injuries were defined as any musculoskeletal condition requiring time away from dancing [29]. They found no association between GJH and a higher injury risk. In addition, Armstrong and colleagues (2019) concluded that a total Beighton score (mean) was a weak predictor of injuries resulting in absence from dancing for one or more days (time-loss) [11]. In contrast, a prospective study of Bronner et al. found that low (0–2) and high (5–9) Beighton scores were significant predictors for medical attention injuries (injuries evaluated by a physical therapist) and time-loss injuries (subset of medical attention injury that involved one or more days of time-loss from dancing following the event) [16].

### Strengths and Limitations of the Study

The first major strength of this study was the high participation rate, low drop-out rate, and high response rate to the monthly questionnaires. This might be explained by two reasons. The high response rate resulted in a large sample size (*N* = 185), enabling us to include multiple factors into the regression models without violation of the commonly used “rule of 10” [30].

The second strength is the use of a prospective cohort design. Several studies have investigated the association between GJH and injuries using a cross-sectional design [13,14]. This design is limited to data at one time point, and can only be applied to study associations. Prospective designs are typically ranked higher in the hierarchy of evidence than cross-sectional designs. In prospective designs, study samples are followed over time, enabling us to investigate a clear sequential relationship (i.e., causality) between an exposure, which happens first, and an outcome, which happens after [31].

The third strength of our study is the use of a broad injury definition (all complaints, substantial injuries, and time-loss injuries). Previous research has shown that the amount of dance students’ injuries and their duration vary depending on the injury definition [24]. Therefore, for every injury definition, we conducted a separate regression analysis. In addition, multiple measurements of GJH were included in the analyses each time. By taking into account broad injury definitions and multiple GJH cut-off points, we analyzed the association between GJH and injury risk to the full extent.

However, there are also some limitations. First of all, in our study, all injuries were self-reported. Most dance students lack medical expertise. Therefore, detailed diagnostic information on registered injuries was missing. This limited us to distinguish between diagnoses of different injury types (for example, acute versus overuse injuries).

Secondly, we asked the student to provide injury information on a monthly basis. This long recall period may have led to underestimation of injuries. Although it is recommended to register injuries on a weekly basis, the literature shows that the average prevalence of injuries was not affected by the sampling frequency up to a frequency of one sample every 4 weeks [20]. Therefore, we do not believe that our recall period of 4 weeks has influenced the reliability of the injury registration. Finally, in the current study, health problems were assessed with the OSTRC questionnaire [20]. Since data collection finished, an update of the OSTRC questionnaire has been published in which changes to the wording, structure, and logic were introduced [32]. We do not believe that using the updated version of the OSTRC questionnaire would have resulted in different findings, because Clarsen and colleagues showed that the number of substantial health problems captured with both versions of the OSTRC questionnaire largely overlap [32].

## 5. Conclusions and Implications for Practice

Healthcare professionals involved in the care for pre-professional dancers should be aware of the increased injury risk and high hypermobility scores in this specific population. The Beighton score has frequently been used in practice to assess GJH and its relation to injury risk in dancers [11,16,29]. The results of the present study show that this test is not conclusive enough to define which dancers are prone to injuries. This may not be a surprise, because the literature states that there is no screening test with adequate test properties to predict injuries [33]. Our findings suggest that dancers with a history of injuries are at higher risk for sustaining a new injury (defined as all complaint injuries, substantial injuries, and time-loss injuries). This is supported by several other publications [3,16]. Therefore, we believe that health professionals should incorporate a thorough examination of the injury history in the physical examination of pre-professional dancers. The explained variance of our models was low, therefore, further research is needed to gain more insight into risk factors in order to develop preventive strategies.

## Figures and Tables

**Table 1:** Baseline characteristics of the total study population and separated for the Bachelor Dance program and the Bachelor Dance teacher program.

	Total Population (*N* = 185)	Dance (*n* = 117)	Dance Teacher (*n* = 68)
Sex (female)	127 (68.6%)	63 (53.8%)	64 (94.1%)
Age (years)	19.1 ± 1.3	18.8 ± 0.9	19.4 ± 1.9
Previous long-lasting injury (yes) ^#^	60 (33.3%)	29 (25.9%)	31 (45.6%)
Generalized joint hypermobility *			
Beighton score	2.8 ± 2.3	3.2 ± 2.5	2.2 ± 2.0
Not hypermobile (0–3) Hypermobile (4–6) Extreme hypermobile (7–9)	129 (70.9%) 25 (13.7%) 28 (15.4%)	76 (65.5%) 17 (14.7%) 23 (19.8%)	53 (80.3%) 8 (12.1%) 5 (7.6%)
Not hypermobile (0–3) Hypermobile (4–9)	129 (70.9%) 53 (29.1%)	76 (65.5%) 40 (34.5%)	53 (80.3%) 13 (19.7%)
Not hypermobile (0–5) Hypermobile (6–9)	154 (84.6%) 28 (15.4%)	93 (80.2%) 23 (19.8%)	61 (92.4%) 5 (7.6%)

Data are presented as mean (SD) or number (%). ^#^ missing data of *n* = 5 (5 dance), * missing data of *n* = 3 (1 Dance, 2 Dance teacher).

**Table 2:** Univariate and multivariate models of potential risk factors for all complaints injuries.

	Non-Injured (*n* = 60)	Injured (*n* = 125)	Univariate Analysis OR (95% CI)	Multivariate Analysis OR (95% CI)			
Sex (female)	44 (73.3%)	83 (66.4%)	0.72 (0.36–1.42)	0.75 (0.33–1.70)	0.65 (0.29–1.49)	0.67 (0.30–1.53)	0.71 (0.32–1.56)
Age (years)	19.0 ± 1.15	19.1 ± 1.43	1.08 (0.85–1.39)	1.04 (0.80–1.35)	1.04 (0.80–1.35)	1.04 (0.80–1.35)	1.04 (0.80–1.35)
Educational Program							
Bachelor dance Bachelor dance teacher	37 (61.7%) 23 (38.3%)	80 (64.0%) 45 (36.0%)	Ref. 0.91 (0.48–1.71)	Ref. 0.84 (0.37–1.88)	Ref. 0.93 (0.42–2.06)	Ref. 0.94 (0.42–2.07)	Ref. 0.88 (0.40–1.92)
Previous long-lasting injury (yes) ^#^	12 (20.3%)	48 (39.7%)	**2.58 (1.24–5.35)**	**2.74 (1.26–5.94)**	**2.69 (1.24–5.82)**	**2.60 (1.21–5.59)**	**2.68 (1.24–5.81)**
Generalized joint hypermobility *							
Beighton score (0–9)	2.95 ± 2.23	2.78 ± 2.40	0.97 (0.85–1.11)	0.96 (0.82–1.12)			
Not hypermobile (0–3) Hypermobile (4–6) Extreme hypermobile (7–9)	41 (71.9%) 7 (12.3%) 9 (15.8%)	88 (70.4%) 18 (14.4%) 19 (15.2%)	Ref. 1.20 (0.46–3.09) 0.98 (0.41–2.36)		Ref. 1.42 (0.51–3.90) 0.94 (0.35–2.49)		
Not hypermobile (0–3) Hypermobile (4–9)	41 (71.9%) 16 (28.1%)	88 (70.4%) 37 (29.6%)	Ref. 1.08 (0.54–2.16)			Ref. 1.15 (0.53–2.49)	
Not hypermobile (0–5) Hypermobile (6–9)	48 (84.2%) 9 (15.8%)	106 (84.8%) 19 (15.2%)	Ref. 0.96 (0.40–2.27)				Ref. 0.87 (0.34–2.27)
Nagelkerke R^2^				0.059	0.060	0.057	0.057

Data are presented as mean (SD) or number (%). ^#^ missing data of *n* = 5 (5 students of Bachelor of Dance), * missing data of *n* = 3 (1 student of Bachelor of Dance, 2 students of Bachelor of Dance Teacher), Ref. = reference; OR = odds ratio; CI = confidence interval. Bold, significant associations.

**Table 3:** Univariate and multivariate models of potential risk factors for substantial injuries.

	Non-Injured (n = 105)	Injured (n = 80)	Univariate Analysis OR (95% CI)	Multivariate Analysis OR (95% CI)			
Sex (female)	69 (65.7%)	58 (72.5%)	1.38 (0.73–2.60)	1.14 (0.52–2.50)	0.97 (0.44–2.15)	1.01 (0.46–2.21)	1.04 (0.49–2.22)
Age (years)	19.04 ± 1.27	19.08 ± 1.44	1.02 (0.82–1.27)	0.95 (0.75−1.20)	0.95 (0.75–1.20)	0.95 (0.75–1.20)	0.95 (0.75–1.20)
Educational Program							
Bachelor dance Bachelor dance teacher	73 (69.5%) 32 (30.5%)	44 (55.0%) 36 (45.0%)	Ref. **1.87 (1.02–3.42)**	Ref. 1.57 (0.73–3.39)	Ref. 1.78 (0.83–3.82)	Ref. 1.79 (0.84–3.83)	Ref. 1.70 (0.81–3.58)
Previous long-lasting injury (yes) ^#^	23 (22.3%)	37 (48.1%)	**3.22 (1.69–6.13)**	**2.96 (1.50–5.84)**	**2.85 (1.45–5.61)**	**2.76 (1.42–5.37)**	**2.84 (1.45–5.59)**
Generalized joint hypermobility *							
Beighton score (0–9)	2.92 ± 2.38	2.71 ± 2.31	0.96 (0.85–1.09)	0.94 (0.81–1.08)			
Not hypermobile (0–3) Hypermobile (4–6) Extreme hypermobile (7–9)	73 (71.6%) 13 (12.7%) 16 (15.7%)	56 (70.0%) 12 (15.0%) 12 (15.0%)	Ref. 1.20 (0.51–2.84) 0.98 (0.43–2.23)		Ref. 1.33 (0.51–3.43) 0.90 (0.35–2.27)		
Not hypermobile (0–3) Hypermobile (4–9)	73 (71.6%) 29 (28.4%)	56 (70.0%) 24 (30.0%)	Ref. 1.08 (0.57–2.05)			Ref. 1.08 (0.52–2.25)	
Not hypermobile (0–5) Hypermobile (6–9)	86 (84.3%) 16 (15.7%)	68 (85.0%) 12 (15.0%)	Ref. 0.95 (0.42–2.14)				Ref. 0.84 (0.34–2.10)
Nagelkerke R^2^				0.115	0.113	0.110	0.110

Data are presented as mean (SD) or number (%). ^#^ missing data of *n* = 5 (5 students of Bachelor of Dance), * missing data of *n* = 3 (1 student of Bachelor of Dance, 2 students of Bachelor of Dance Teacher), Ref. = reference; OR = odds ratio; CI = confidence interval. Bold, significant associations.

**Table 4:** Univariate and multivariate models of potential risk factors for time-loss injuries.

	Non-Injured (*n* = 84)	Injured (*n* = 101)	Univariate Analysis OR (95% CI)	Multivariate Analysis OR (95% CI)			
Sex (female)	60 (71.4%)	67 (66.3%)	0.79 (0.42–1.48)	0.65 (0.30–1.39)	0.60 (0.28–1.31)	0.60 (0.28–1.31)	0.62 (0.29–1.30)
Age (years)	18.94 ± 1.10	19.15 ± 1.51	1.13 (0.90–1.42)	1.05 (0.82–1.35)	1.05 (0.82–1.35)	1.05 (0.82–1.35)	1.05 (0.82–1.35)
Educational Program							
Bachelor dance Bachelor dance teacher	57 (67.9%) 27 (32.1%)	60 (59.4%) 41 (40.6%)	Ref. 1.44 (0.79–2.65)	Ref. 1.46 (0.68 −3.15)	Ref. 1.57 (0.73–3.36)	Ref. 1.57 (0.73–3.36)	Ref. 1.54 (0.73–3.26)
Previous long-lasting injury (yes) ^#^	17 (20.5%)	43 (44.3%)	**3.09 (1.59–6.02)**	**3.04 (1.50–6.15)**	**2.95 (1.46–5.94)**	**2.94 (1.47–5.89)**	**2.94 (1.46–5.93)**
Generalized joint hypermobility *							
Beighton score (0–9)	2.94 ± 2.29	2.74 ± 2.39	0.97 (0.85–1.09)	0.97 (0.84–1.13)			
Not hypermobile (0–3) Hypermobile (4–6) Extreme hypermobile (7–9)	57 (70.4%) 12 (14.8%) 12 (14.8%)	72 (71.3%) 13 (12.9%) 16 (15.8%)	Ref. 0.86 (0.36–2.02) 1.06 (0.46–2.41)		Ref. 1.10 (0.43–2.83) 1.09 (0.43–2.75)		
Not hypermobile (0–3) Hypermobile (4–9)	57 (70.4%) 24 (29.6%)	72 (71.3%) 29 (28.7%)	Ref. 0.96 (0.50–1.82)			Ref. 1.09 (0.53–2.27)	
Not hypermobile (0–5) Hypermobile (6–9)	69 (85.2%) 12 (14.8%)	85 (84.2%) 16 (15.8%)	Ref. 1.08 (0.48–2.44)				Ref. 1.07 (0.43–2.64)
Nagelkerke R^2^				0.099	0.098	0.098	0.098

Data are presented as mean (SD) or number (%). ^#^ missing data of *n* = 5 (5 students of Bachelor of Dance), * missing data of *n* = 3 (1 student of Bachelor of Dance, 2 students of Bachelor of Dance Teacher), Ref. = reference; OR = odds ratio; CI = confidence interval. Bold, significant associations.

## Data Availability

The data presented in this study are available on request from the corresponding author. The data are not publicly available due to privacy.
